# Adaptive Random Testing with Combinatorial Input Domain

**DOI:** 10.1155/2014/843248

**Published:** 2014-03-19

**Authors:** Rubing Huang, Jinfu Chen, Yansheng Lu

**Affiliations:** ^1^School of Computer Science and Telecommunication Engineering, Jiangsu University, 301 Xuefu Road, Zhenjiang, Jiangsu 212013, China; ^2^School of Computer Science and Technology, Huazhong University of Science and Technology, Wuhan, Hubei 430074, China

## Abstract

Random testing (RT) is a fundamental testing technique to assess software reliability, by simply selecting test cases in a random manner from the whole input domain. As an enhancement of RT, adaptive random testing (ART) has better failure*-*detection capability and has been widely applied in different scenarios, such as numerical programs, some object*-*oriented programs, and mobile applications. However, not much work has been done on the effectiveness of ART for the programs with combinatorial input domain (i.e., the set of categorical data). To extend the ideas to the testing for combinatorial input domain, we have adopted different similarity measures that are widely used for categorical data in data mining and have proposed two similarity measures based on interaction coverage. Then, we propose a new version named ART*-*CID as an extension of ART in combinatorial input domain, which selects an element from categorical data as the next test case such that it has the lowest similarity against already generated test cases. Experimental results show that ART*-*CID generally performs better than RT, with respect to different evaluation metrics.

## 1. Introduction

Software testing, a major software engineering activity, is widely considered to assure the quality of software under test [1]. Many testing methods have been developed to effectively identify software failures by actively selecting inputs (namely, test cases). Random testing (RT), a basic software testing method, simply chooses test cases at random from the set of all possible program inputs (namely, the input domain) [2, 3]. There are many advantages of using RT in software testing. For example, in addition to simplicity and the efficiency of generating random test cases [2], RT allows statistical quantitative estimation of software's reliability [4]. Due to these advantages, RT has been widely used to detect software failures in different scenarios, such as the testing of UNIX utilities [5, 6], SQL database systems [7, 8], Java JIT compilers [9], and embedded software systems [10]. In spite of the popularity, RT is still criticized by many researchers due to little or no information to guide its test case generation.

Given a faulty program, two basic features are determined by program inputs causing software to exhibit failure behaviors (namely, failure-causing inputs), that is, failure rate *θ* and failure pattern. Failure rate refers to the ratio between the number of failure-causing inputs and the number of all possible program inputs, while failure pattern refers to the geometry and distribution of failure regions (i.e., the region where failure-causing inputs reside). It has been observed, however, that failure-causing inputs tend to cluster together [11–13]. Given that failure regions are continuous, nonfailure regions should also be contiguous. More specifically, suppose a test case (tc) is not a failure-causing input, test cases that are close to tc (or tc's neighbors) may fail to reveal a failure as well. Therefore, it is intuitively appealing that test cases that spread away from tc may have a higher chance to be failure-causing than tc's neighbors.

Briefly speaking, it is very likely that a more even-spread of random test cases can improve the failure-detection effectiveness of RT. Based on this intuition, Chen et al. [14] have proposed a novel approach, namely, adaptive random testing (ART). Similar to RT, ART also randomly generates test case from the whole input domain. But ART uses additional criteria to guide the test case selection for the purpose of evenly spreading test cases over the input domain. Various ART algorithms have been developed based on different test case selection criteria, such as ART by distance [15], ART by exclusion [16], ART based on evolutionary search algorithms [17], and ART by perturbation [18]. Essentially, ART achieves test case diversity with the subset of test cases executed at any one time [19].

As an alternative of RT, ART has been successfully applied to different programs, such as numerical programs [15–18], object-oriented programs [20, 21], and mobile application [22]. However, not much work has been done on the effectiveness of ART for programs with combinatorial input domain (or categorical data, i.e., a Cartesian product of finite value domains for each of a finite set of parameter variables). With the popularity of category-partition method [23] and many guidelines to help construct categories and partitions [24–27], combinatorial input domain has been widely applied to different testing scenarios, such as configurable-aware system [28, 29], event-driven software [30], and GUI-based application [31]. In this paper, we propose a new testing strategy called ART-CID as an extension of ART in combinatorial input domain. In order to successfully extend the ART principle into combinatorial input domain, we propose two similarity measures based on interaction coverage and also adopt different well-studied similarity measures that are popularly used for categorical data in data mining [32]. To analyze the effectiveness of ART-CID (mainly FSCS-CID, one version of ART-CID), we compare the effectiveness of FSCS-CID with RT by designing some simulations and the empirical study. Experimental results show that, compared with RT, FSCS-CID can not only use smaller test cases in order to cover all possible combinations of parameter values at a given strength, but also require to generate fewer test cases to identify the first failure in the real-life program.

This paper is organized as follows.  Section 2 introduces some preliminaries, including combinatorial input domain, ART, similarity measures used for combinatorial input domain, and the effectiveness measures adopted in our study.  Section 3 proposes two similarity measures for combinatorial test cases based on interaction coverage.  Section 4 proposes a new algorithm called ART-CID to select test cases from combinatorial input domain.  Section 5 reports some experimental studies, which examine the rate of covering value combinations at a given strength and failure-detection effectiveness of our new method. Finally, Section 6 summarizes some discussions and conclusions.

## 2. Preliminaries

In the following section, some preliminaries of combinatorial input domain, failure patterns, adaptive random testing, similarity and dissimilarity measures for combinatorial input domain, and effectiveness measure are described.

### 2.1. Combinatorial Input Domain

Suppose that a system under test (SUT) has a set of *k* parameters (or categories) *P* = {*p*_1_, *p*_2_,…, *p*_*k*_}, which may represent user inputs, configuration parameters, internal events, and so forth. Let *V*_*i*_ be the finite set of discrete valid values (or choices) for *p*_*i*_ (*i* = 1,2,…, *k*), and let *C* be the set of constraints on parameter value combinations. Without loss of generality, we assume that the order of parameters is fixed; that is, *P* = 〈*p*_1_, *p*_2_,…, *p*_*k*_〉. In the remainder of this paper, we will refer to a* combination of parameters* as a* parameter interaction*, and a* combination of parameter values* or a* parameter value combination* as a* value combination*.

**Definition 1**

A test profile, denoted as TP(*k*, |*V*_1_ | |*V*_2_ | ⋯|*V*_*k*_ | , *C*), is about the information on a combinatorial input domain of the SUT, including *k* parameters, |*V*_*i*_| (*i* = 1,2,…, *k*) values for parameter *p*_*i*_, and constraints *C* on value combinations.

In this paper, we assume that all the parameters are independent; that is, no constraint among value combinations is considered (*C* = *∅*), unless otherwise specified. Therefore, the test profile can be abbreviated as TP(*k*, |*V*_1_ | |*V*_2_ | ⋯|*V*_*k*_|).

To clearly describe some notions and definitions, we present an example of the part of suboptions in an option “View” of the tool PDF shown in Table 1. In this system, there are four configuration parameters, each of which has three values. Therefore, its test profile can be written as TP(4, 3^4^).

**Definition 2**

Given a TP(*k*, |*V*_1_ | |*V*_2_ | ⋯|*V*_*k*_|), a test case or a test configuration is a *k*-tuple (*v*_1_, *v*_2_,…, *v*_*k*_) where *v*_*i*_ ∈ *V*_*i*_ (*i* = 1,2,…, *k*).

Intuitively speaking, a combinatorial input domain TP(*k*, |*V*_1_ | |*V*_2_ | ⋯|*V*_*k*_|) is a Cartesian product of 〈*V*_1_, *V*_2_,…, *V*_*k*_〉 for each of *P*; that is, *T*_all_ = *V*_1_ × *V*_2_ × ⋯×*V*_*k*_. Therefore, the size of all possible test cases is |*V*_1_ | ×|*V*_2_ | ×⋯×|*V*_*k*_|. For example, a 4-tuple tc = (*Full* *Size*, *Single*, *Clockwise*, *All* *Pages*) is a test case for the SUT shown in Table 1.

**Definition 3**

Given a TP(*k*, |*V*_1_ | |*V*_2_ | ⋯|*V*_*k*_|), a *τ*-wise value combination is a *k*-tuple $\left({\hat{v}}_{1},{\hat{v}}_{2},\ldots ,{\hat{v}}_{k}\right)$ involving *τ* parameters with fixed values (named fixed parameters) and (*k* − *τ*) parameters with arbitrary allowable values (named free parameters), where 0 ≤ *τ* ≤ *k* and

(1) $$\begin{array}{c} {\hat{v}}_{i}=\{\begin{array}{cc} v_{i}\in V_{i}, & \text{if}\;p_{i}\;\text{is}\;\text{a}\;\text{fixed}\;\text{parameter};\\ \;“-\text{"}, & \text{if}\;p_{i}\;\text{is}\;\text{a}\;\text{free}\;\text{parameter}. \end{array} \end{array}$$

Generally, *τ*-wise value combination is also called *τ*-value schema [33], and *τ* is called strength. When *τ* = *k*, a *τ*-wise value combination becomes a test case for the SUT as it takes on a specific value for each of its parameters. For ease of description, we define a term CombSet_*τ*_(tc) as the set of *τ*-wise value combinations covered by the test case (tc). Intuitively speaking, a test case (tc) with *k* parameters contains *C*_*k*_^*τ*^ *τ*-wise value combinations, that is, |CombSet_*τ*_(tc)| = *C*_*k*_^*τ*^.

For example, considering a test case (tc) = (*Full* *Size*, *Single*, *Clockwise*, *All* *Pages*), we can obtain that CombSet_1_(tc) = {(*Full* *Size*, −, −, −), (−, *Single*, −, −), (−, −, *Clockwise*, −), (−, −, −, *All* *Pages*)}, while CombSet_3_(tc) = {(*Full* *Size*, *Single*, *Clockwise*, −), (*Full* *Size*, *Single*, −, *All* *Pages*), (*Full* *Size*, −, *Clockwise*, *All* *Pages*), (−, *Single*, *Clockwise*, *All* *Pages*)}.

**Definition 4**

The number of parameters required to trigger a failure is referred to as the failure-triggering fault interaction (FTFI) number.

As we know, the faulty model in the combinatorial input domain assumes that failures are caused by parameter interactions. For instance, if the SUT shown in Table 1 fails when *p*_2_ is set to “*Single*”, *p*_3_ is set to “*None,*” and *p*_4_ is not equal to “*None,*” this failure is caused by the parameter interaction (*p*_2_, *p*_3_, *p*_4_). Therefore, the FTFI number of this fault is 3.

In [28, 34], Kuhn et al. investigated interaction failures by analyzing the faults reports of several software projects and concluded that failures are always caused by low FTFI numbers.

### 2.2. Failure Patterns

Given a faulty program, two basic features can be obtained from it. One feature is* failure rate*, denoted by *θ*, which refers to the ratio of the number of failure-causing inputs to the number of all possible inputs. The other feature is* failure pattern*, which refers to the geometric shapes and the distributions of the failure-causing regions. Both features are fixed but unknown to testers before testing.

**Figure 1:**
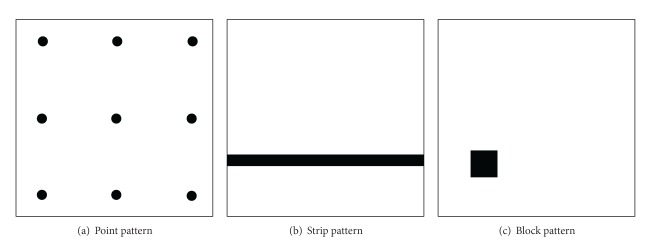
Three types of failure patterns with two-dimensional input domain.

In [14], the patterns of failure-causing inputs have been classified into three categories:* point pattern*,* stripe pattern,* and* block pattern*. An illustrative example about three types of failure patterns in a two-dimensional input domain is shown in Figure 1. In this example, suppose the input domain is consisting of parameters *x* and *y* where 0 ≤ *x*, *y* ≤ 10.0. Point pattern means the tested program will fail when *x* and *y* are assigned to particular integers, that is, some specific points in the input domain, while strip pattern may be of the form 0 ≤ *x* ≤ 10.0, 3.0 ≤ *y* ≤ 3.5, and block pattern may be of the form 1.5 ≤ *x*, *y* ≤ 3.0.

In the combinatorial input domain, failure patterns of any failures belong to the point pattern as all test inputs are discrete. However, from the perspective of functionality and computation of each test input, three failure patterns shown in Figure 1 also exist in the combinatorial input domain. For example, (1) if a failure *f*_1_ in the SUT shown in the Table 1 is caused by “*p*_2_ = *Single* or *p*_2_ = *Continuous*” and “*p*_3_ = *Clockwise* or *p*_3_ = *None*”, we believe that the failure pattern of *f*_1_ is a strip pattern and its failure rate is (2 × 2)/(3 × 3) = 0.4444; (2) if a failure *f*_2_ in the SUT is caused by “*p*_1_ ≠ *Full* *Size*”, “*p*_2_ ≠ *Single*”, “*p*_3_ ≠ *None*”, and “*p*_4_ ≠ *All* *pages*”, we believe that the failure pattern of *f*_2_ is a block pattern and its failure rate is (2/3)^4^ = 0.1975; and (3) if a failure *f*_3_ is caused by a single test case (*Full* *Size*, *Single*, *Clockwise*, *None*), we believe that the failure region of *f*_3_ is a point pattern and its failure rate is 1/81 = 0.0123. According to Kuhn's investigations [28, 34], however, the FTFI numbers are always very low (i.e., the FTFI numbers are smaller than the number of parameters), which means that the strip pattern is the most frequent failure pattern in the combinatorial input domain.

### 2.3. Adaptive Random Testing (ART)

The methodology of adaptive random testing (ART) [14, 15] has been proposed to enhance the failure-detection effectiveness of random testing (RT) by even-spreading test cases across the whole input domain. In ART, test cases are not only randomly generated, but also evenly spread. According to previous ART studies [15–22], ART was shown to reduce the number of test cases required to identify the first fault by as much as 50% over RT.

There are many implementations of ART by different notions. A simple algorithm is the fixed-size-candidate-set ART (FSCS-ART) [15]. FSCS-ART implements the notion of distance as follows. FSCS-ART uses two sets of test cases, namely, the executed set *E* and the candidate set *C*. *E* is a set of test cases that have been executed but without revealing any failure, while *C* is a set of tests that are randomly selected from the input domain according to the uniform distribution. *E* is initially empty and the first element is randomly chosen from the input domain and then incrementally updates with the selected elements from *C* until a failure is exhibited. From *C*, the element that is farthest away from all test cases in *E* is chosen as the next test case; that is, the criterion is to choose the element *c*′ from *C* as the next test case such that

(2) $$\begin{array}{c} \begin{array}{c} \left(\forall c\right)\left(c\in C\right)\left(c\neq c^{\prime }\right)\left[\underset{e\in E}{\min}\mathrm{dist}\left(c^{\prime },e\right)\geq \underset{e\in E}{\min}\mathrm{dist}\left(c,e\right)\right], \end{array} \end{array}$$

where dist is defined as the Euclidean distance, that is, in a *m*-dimensional input domain, for two test inputs, tc_1_ = (*a*_1_, *a*_2_,…, *a*_*m*_) and tc_2_ = (*b*_1_, *b*_2_,…, *b*_*m*_),

(3) $$\begin{array}{c} \begin{array}{c} \mathrm{dist}\left(\text{t}{\text{c}}_{1},\text{t}{\text{c}}_{2}\right)=\sqrt{\overset{m}{\underset{i=1}{\sum }}{\left(a_{i}-b_{i}\right)}^{2}}. \end{array} \end{array}$$

The process is repeated until the desired stopping criterion is satisfied.

**Figure 2:**
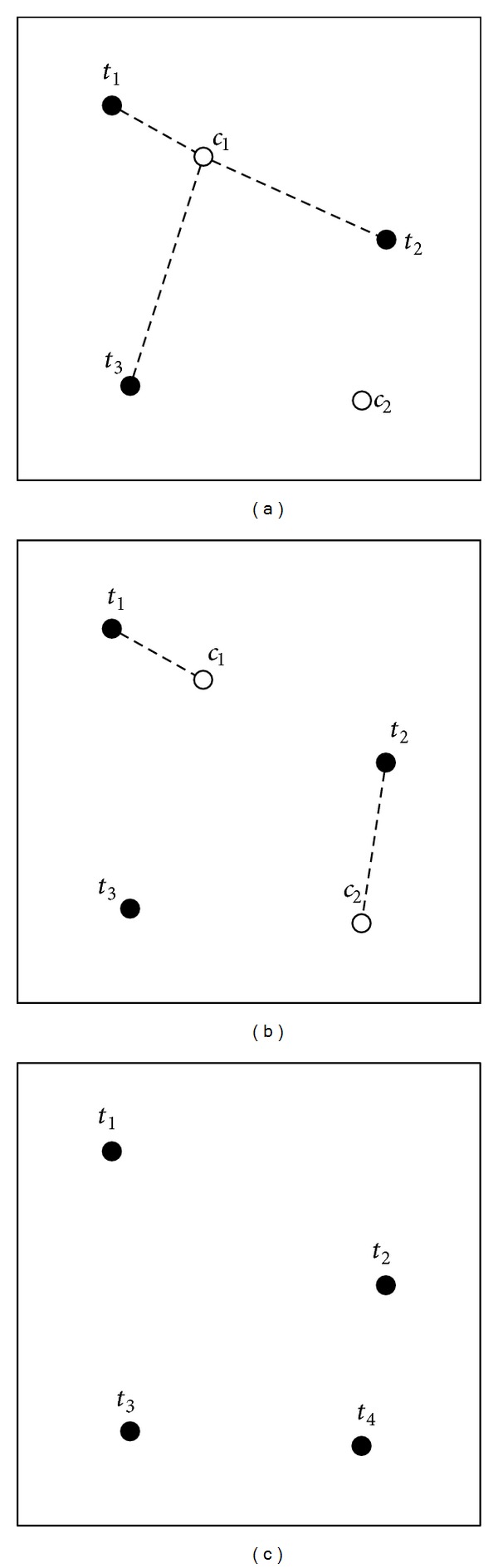
Illustration of FSCS-ART in a two-dimensional input domain. Previously executed test cases are denoted as *t*_1_, *t*_2_, and *t*_3_, and randomly generated candidates are denoted as *c*_1_ and *c*_2_, respectively. To select the next test case, (a) multiple candidates are randomly selected, and the nearest previously executed test case to each candidate is determined; (b) these nearest distances are compared among all candidates; and (c) the candidate with the longest such distance is chosen.

Figure 2 gives the illustration of FSCS-ART in a two-dimensional input domain. In Figure 2(a), there are 3 previously executed test cases *t*_1_, *t*_2_, and *t*_3_, and 2 randomly generated candidates *c*_1_ and *c*_2_. To choose among the candidates, the distance of each candidate against each previously executed test case is calculated.  Figure 2(b) describes that the closest previously executed test case is determined for each candidate. In Figure 2(c), the candidate *c*_2_ is selected as the next test case (i.e., *t*_4_ = *c*_2_), as the distance of *c*_2_ against its nearest previously executed test case is larger than that of the candidate *c*_1_.

In this paper, we emphasize the extension of FSCS-ART as that of ART in combinatorial input domain, unless otherwise specified.

### 2.4. Similarity and Dissimilarity Measures for Combinatorial Input Domain

Measuring similarity or dissimilarity (distance) between two test inputs is a core requirement for test case selection, evaluation, and generation. Generally speaking, in numerical input domains, Euclidean distance (see (3)) is a mostly used distance measure for continuous data. However, for a combinatorial input domain, since its parameters and corresponding values are finite and discrete, Euclidean distance may not be available and reasonable. Nevertheless, various distance measures (or dissimilarity measures) are popularly used in data mining for evaluating categorical data [32], such as clustering (*k*-means), classification (KNN, SVM), and distance-based outlier detection. In this subsection, we simply describe the following measures that will be adopted in our paper later.

To illustrate our work clearly, let us define a few terms. Consider a categorical dataset *T* containing *N* objects, derived from a TP(*k*, |*V*_1_ | |*V*_2_ | ⋯|*V*_*k*_|) for parameters *P*_1_, *P*_2_,…, *P*_*k*_. We also use the following notation.

(i) *f*_*i*_(*x*) is the number of times parameter *P*_*i*_ takes the value *x* in *T*. Note that if *x* ∉ *V*_*i*_, *f*_*i*_(*x*) = 0.
(ii) ${\hat{p}}_{i}(x)$ is the sample probability of parameter *P*_*i*_ to take the value *x* in *T*. The sample probability is given by
  (4) $$\begin{array}{c} {\hat{p}}_{i}\left(x\right)=\frac{f_{i}\left(x\right)}{N}. \end{array}$$
(iii) *p*_*i*_^2^(*x*) is another probability estimate of parameter *P*_*i*_ to take the value *x* in *T* and is given by
  (5) $$\begin{array}{c} p_{i}^{2}\left(x\right)=\frac{f_{i}\left(x\right)\left(f_{i}\left(x\right)-1\right)}{N\left(N-1\right)}. \end{array}$$
(iv) *S*(*X*, *Y*) is a generalized similarity measure between two data instances denoted as *X* = (*X*_1_, *X*_2_,…, *X*_*k*_) and *Y* = (*Y*_1_, *Y*_2_,…, *Y*_*k*_) where *X*, *Y* ∈ *T*, and *X*_*i*_, *Y*_*i*_ ∈ *V*_*i*_ (*i* = 1,2,…, *k*). Its definition is given as follows:
  (6) $$\begin{array}{c} S\left(X,Y\right)=\overset{k}{\underset{i=1}{\sum }}w_{i}S_{i}\left(X_{i},Y_{i}\right), \end{array}$$

where *S*_*i*_(*X*_*i*_, *Y*_*i*_) (*i* = 1,2,…, *k*) is the per-parameter similarity between two values for parameter *P*_*i*_ and *w*_*i*_ denotes the weight assigned to the parameter *P*_*i*_. Therefore, we only require to present the definitions of *S*_*i*_(*X*_*i*_, *Y*_*i*_) and *w*_*i*_ for each similarity measure, unless otherwise specified.

To directly refer to [32], the measures discussed henceforth will all be in the context of similarity, with dissimilarity or distance measures being converted using the following formula:

(7) $$\begin{array}{c} S\left(X,Y\right)=\frac{1}{1+D\left(X,Y\right)}, \end{array}$$

where *D*(*X*, *Y*) is the dissimilarity measure between *X* and *Y*.

Table 2 presents nine similarity measures for categorical parameter values, which are widely used in data mining for categorical data. In Table 2, the last column “Range” represents the range of *S*_*i*_(*X*_*i*_, *Y*_*i*_) for mismatches or matches of parameter values in each measure.

### 2.5. Effectiveness Measurement

In this paper, we adopt the *F*-*measure* (i.e., the number of test cases required to detect the first failure) as the measurement of failure-detection effectiveness of testing methods, since previous studies [35] have demonstrated that the *F*-measure is particularly suitable for adaptive testing strategies such as ART. Intuitively speaking, a smaller *F*-measure of ART over RT means fewer test cases required by ART to detect the first failure and hence implies a better failure-detection effectiveness of ART than that of RT. For the purpose of clear description, we will use ART *F*-*ratio* (i.e., the ratio of ART's *F*-measure (*F*_ART_) relative to RT's *F*-measure (*F*_RT_)) to indicate the failure-detection effectiveness improvement of ART over RT.

However, it is extremely difficult to theoretically obtain ART's *F*-measure (*F*_ART_). Similar to all other ART studies, *F*_ART_ is collected via simulations and empirical studies, whose procedure is described as follows. On the one hand, in simulation studies, failure pattern (including its size and sharp) and failure rate *θ* are predefined for simulating a faulty program. The failure regions are then randomly placed inside the whole input domain. If a point inside one of the failure regions is picked by a testing strategy, a failure is said to be detected. On the other hand, for empirical studies, some faults are seeded into a subject program. Once the subject program behaves differently from its fault-seeded version, it is said that a failure is identified. The number of test cases to find the first failure is regarded as the *F*_ART_ of that run. Such a process runs *𝒮* times repeatedly until a statistically reliable estimate of the *F*_ART_ (±5% accuracy rate and (1 − 5%) confidence level adopted in our paper) has been obtained. Refer to the value of *𝒮*; it can be determined dynamically using the same method as shown in [15]. With respect to RT's *F*-measure (*F*_RT_), since test cases are chosen with replacement according to the uniform distribution, *F*_RT_ is equal to 1/*θ* theoretically.

Apart from the *F*-measure used as the measurement, another measurement is also used in our paper, that is, the number of test cases required to first cover all possible value combinations of a given strength *τ* (denoted *N*_*τ*_-measure). This measurement is widely used in the combinatorial input domain. Unlike the *F*-measure, the testing stop condition of *N*_*τ*_-measure is not that the first failure is detected, but that all possible *τ*-wise value combinations are first covered. For the purpose of clear description, we use *N*_RT_(*τ*) to represent this measurement for RT while *N*_ART_(*τ*) for ART.

## 3. Two Similarity Measures Based on Interaction Coverage

Apart from various similarity measures described in Section 2.4, in this section, we propose another two similarity measures by using interaction coverage: incremental interaction coverage similarity (IICS) and multiple interaction coverage similarity (MICS), in order to apply the characteristics of combinatorial input domain to the selection of test cases. All similarity measures illustrated in Section 2.4 are used to evaluate how similar two test cases are; however, two similarity measures presented in this section are used to evaluate the resemblance of the combinatorial test case against the combinatorial test suite. We will discuss them next.

Before introducing them, we firstly describe a simple similarity measure of the test case against a test suite based on interaction coverage, named normalized covered *τ*-wise value combinations similarity (or NCVCS_*τ*_) [36], which is widely used in combinatorial input domain.

**Definition**

Given a combinatorial test suite *T* on TP(*k*, |*V*_1_ | |*V*_2_ | ⋯|*V*_*k*_|), a combinatorial test case (tc), and the strength *τ*, normalized covered *τ*-wise value combinations similarity (NCVCS_*τ*_) of tc against *T* is defined as the ratio of the number of *τ*-wise value combinations covered by tc that have already been covered by *T* to *C*_*k*_^*τ*^; that is,

(8) $$\begin{array}{c} \text{NCVC}{\text{S}}_{\tau }\left(\text{tc},T\right)=\frac{\left|\text{CombSe}{\text{t}}_{\tau }\left(\text{tc}\right)⋂\text{CombSe}{\text{t}}_{\tau }\left(T\right)\right|}{C_{k}^{\tau }}, \end{array}$$

where CombSet_*τ*_(*T*) can be written as follows:

(9) $$\begin{array}{c} \text{CombSe}{\text{t}}_{\tau }\left(T\right)=\underset{\text{tc}\in T}{⋃}\text{CombSe}{\text{t}}_{\tau }\left(\text{tc}\right). \end{array}$$

Obviously, the NCVCS_*τ*_ is a function that requires to set the strength value *τ* in advance, and its range is [0,1.0]. Two properties of the NCVCS_*τ*_ are discussed as follows.

**Theorem 6**

If *NCVCS*_*τ*_(*tc*, *T*) = 1.0, *NCVCS*_*λ*_(*tc*, *T*) = 1.0, where 1 ≤ *λ* < *τ* ≤ *k*.

**Proof**

When NCVCS_*τ*_(tc, *T*) = 1.0, it can be noted that *T* covers all possible *τ*-wise value combinations covered by *tc*, that is,

(10) $$\begin{array}{c} \text{CombSe}{\text{t}}_{\tau }\left(\text{tc}\right)\subseteq \text{CombSe}{\text{t}}_{\tau }\left(T\right). \end{array}$$

Since

(11) CombSetλ(tc)=CombSetλ(CombSetτ(tc))⊆CombSetλ(CombSetτ(T))=CombSetλ(T) (1≤λ<τ≤k),

*T* also covers all possible value combinations at strengths lower than *τ* that are covered by tc. As a consequence, NCVCS_*λ*_(tc, *T*) = 1.0 where 1 ≤ *λ* < *τ* ≤ *k*.

**Theorem 7**

If *NCVCS*_*λ*_(*tc*, *T*) = 0, *NCVCS*_*τ*_(*tc*, *T*) = 0 where 1 ≤ *λ* < *τ* ≤ *k*.

**Proof**

When NCVCS_*λ*_(tc, *T*) = 0, it can be noted that each *λ*-wise value combination covered by tc is not covered by *T*, indicating that, for ∀*e*′ ∈ CombSet_*λ*_(tc), *e*′ ∉ CombSet_*λ*_(*T*): that is,

(12) $$\begin{array}{c} \text{CombSe}{\text{t}}_{\lambda }\left(\text{tc}\right)⋂\text{CombSe}{\text{t}}_{\lambda }\left(T\right)=\emptyset . \end{array}$$

Therefore, the problem converts to demonstrating that ∀*e* ∈ CombSet_*τ*_(tc), *e* ∉ CombSet_*τ*_(*T*).

We suppose that ∃*e* such that *e* ∈ CombSet_*τ*_(tc) and *e* ∈ CombSet_*τ*_(*T*), that is,

(13) CombSetλ(CombSetτ(tc))  ⋂CombSetλ(CombSetτ(T))≠∅.

Due to *λ* < *τ*, (13) is equivalent to the equation shown as follows:

(14) $$\begin{array}{c} \text{CombSe}{\text{t}}_{\lambda }\left(\text{tc}\right)⋂\text{CombSe}{\text{t}}_{\lambda }\left(T\right)\neq \emptyset . \end{array}$$

Obviously, (14) is contradictory to (12). Therefore, ∀*e* ∈ CombSet_*τ*_(tc), *e* ∉ CombSet_*τ*_(*T*), which means that NCVCS_*τ*_(tc, *T*) = 0 where 1 ≤ *λ* < *τ* ≤ *k*.

As we know, given a TP(*k*, |*V*_1_ | |*V*_2_ | ⋯|*V*_*k*_|) and the strength *τ*, the number of all possible *τ*-wise value combinations is fixed; that is, |CombSet_*τ*_(*T*_all_)| = ∑_*i*_1_=1_^*k*−*τ*^ ⋯ ∑_*i*_*τ*_=*i*_*τ*−1_+1_^*k*^(|*V*_*i*_1__ | ⋯|*V*_*i*_*τ*__|). In other words, there exists a test case generation method using NCVCS_*τ*_ as the criterion, which can generate a certain number of combinatorial test cases denoted as *T* (*T*⊆*T*_all_) to cover all possible *τ*-wise value combinations. However, if testing with *T* fails to reveal any failures due to no failure-causing inputs in *T*, the next test case generated by this method is, in fact, obtained in a random manner. The main reason is that the NCVCS_*τ*_ of each element in *T*_all_ is equal to 1.0. Therefore, the NCVCS_*τ*_ is not particularly suitable for adaptive testing strategies such as ART. To solve this problem, we propose two similarity measures based on interaction coverage in the following subsections.

### 3.1. Incremental Interaction Coverage Similarity

As discussed in Theorem 6, if all possible *τ*-wise value combinations are covered by a combinatorial test suite *T*, all possible value combinations at strengths lower than *τ* are also covered by *T*. According to this fact, we present a new similarity measure based on interaction coverage, named incremental interaction coverage similarity (IICS).

Given a combinatorial test suite *T* on TP(*k*, |*V*_1_ | |*V*_2_ | ⋯|*V*_*k*_|) and a combinatorial test case (tc), the incremental interaction coverage similarity of tc against *T* is defined as follows:

(15) IICS(tc,T)={1.0,if  tc∈T;NCVCSτ(tc,T),if  tc∉T,

where *τ* satisfies the following properties: (1)  CombSet_*τ*−1_(*T*) = CombSet_*τ*−1_(*T*_all_) and (2)  CombSet_*τ*_(*T*) < ComSet_*τ*_(*T*_all_), where 1 ≤ *τ* ≤ *k* (assume CombSet_0_(*T*) ≡ CombSet_0_(*T*_all_)).

It can be noted that if tc ∈ *T*, the IICS is equal to 1.0 as tc is the same as one of elements in *T*; if tc ∉ *T*, the IICS of tc against *T* is actually equal to the NCVCS_*τ*_ of tc against *T* where *τ* is gradually incremented. More specifically, if *T* covers all possible *i*-wise value combinations and partial (*i* + 1)-wise value combinations occurred in tc, *τ* = *i* + 1. Similar to NCVCS_*τ*_, the range of IICS is also [0,1.0].

Here, we present an example to illustrate IICS. Suppose *T* = {(0,0, 0), (0,1, 1)} on TP(3, 2^3^), tc_1_ = (1,0, 1), and tc_2_ = (1,1, 0), IICS(tc_1_, *T*) = NCVCS_1_(tc_1_, *T*) = 2/*C*_3_^1^ = 0.67 as 1-wise value combinations are not completely covered by *T*, and hence *τ* = 1. Let *T* = *T*⋃{tc_1_}, IICS(tc_2_, *T*) = NCVCS_2_(tc_2_, *T*) = 0/*C*_3_^2^ = 0 as *T* covers all 1-wise value combinations and partial 2-wise value combinations occurred in tc_2_, and hence *τ* = 2.

### 3.2. Multiple Interaction Coverage Similarity

As shown in Section 3.1, the IICS measure begins at strength *τ* = 1, and then update the value of *τ* by (*τ* + 1). In other words, it considers different strength values when evaluating the combinatorial test case against the combinatorial test suite. However, the IICS accounts for each strength value at each time rather than simultaneously considering all strength values. As a consequence, we present another similarity measure based on interaction coverage, named multiple interaction coverage similarity (MICS).

Given a combinatorial test suite *T* on TP(*k*, |*V*_1_ | |*V*_2_ | ⋯|*V*_*k*_|) and a combinatorial test case (tc), the weighted interaction coverage similarity of tc against *T* is defined as follows:

(16) $$\begin{array}{c} \text{MICS}\left(\text{tc},T\right)=\overset{k}{\underset{\tau =1}{\sum }}\left(\omega_{\tau }*\text{NCVC}{\text{S}}_{\tau }\left(\text{tc},T\right)\right), \end{array}$$

where 0 ≤ *ω*_*τ*_ ≤ 1.0 and ∑_*τ*=1_^*k*^ *ω*_*τ*_ = 1.0.

Intuitively speaking, if tc ∈ *T*, MICS(tc, *T*) = 1.0. Similar to IICS, the MICS ranges from 0 to 1.0.

Here, we present an example to explain the definition of MICS. Let *T* = {(0,0, 0,0), (0,1, 1,1)} on TP(4, 2^4^), tc_1_ = (1,0, 1,0), tc_2_ = (0,1, 1,0), and *ω*_1_ = *ω*_2_ = *ω*_3_ = *ω*_4_, MICS(tc_1_, *T*) = (1/4) × ∑_*τ*=1_^4^NCVCS_*τ*_(tc_1_, *T*) = (1/4)×(1/*C*_4_^1^ + 5/*C*_4_^2^ + 3/*C*_4_^3^ + 1/*C*_4_^4^) = 0.71, while MICS(tc_2_, *T*) = (1/4) × ∑_*τ*=1_^4^CVCS_*τ*_(tc_2_, *T*) = (1/4)×(0/*C*_4_^1^ + 2/*C*_4_^2^ + 2/*C*_4_^3^ + 1/*C*_4_^4^) = 0.46.

### 3.3. Properties of Two New Similarity Measures

Some properties of the proposed two similarity measures are discussed in the following subsection.

**Theorem 8**

If *CombSet*_*k*−1_(*T*) = *CombSet*_*k*−1_(*T*_*all*_), for ∀*tc* ∉ *T*, *IICS*(*tc*, *T*) and *MICS*(*tc*, *T*) remain unchanged.

**Proof**

On the one hand, if CombSet_*k*−1_(*T*) = CombSet_*k*−1_(*T*_all_) (i.e., *T* covers all possible (*k* − 1)-wise value combinations), for ∀tc ∉ *T*,

(17) IICS(tc,T)=NCVCSk(tc,T)=|CombSetk(tc)⋂CombSetk(T)|Ckk=|{tc}⋂T|Ckk=0.

On the other hand, if CombSet_*k*−1_(*T*) = CombSet_*k*−1_(*T*_all_) (i.e., *T* covers all possible (*k* − 1)-wise value combinations), ∀tc ∈ *T*_all_, NCVCS_*k*−1_(tc, *T*) = 1.0. According to Theorem 6, it can be concluded that NCVCS_*τ*_(tc, *T*) = 1.0, where 1 ≤ *τ* < *k* − 1; that is, *T* covers all possible *τ*-wise value combinations. In other words, CombSet_*τ*_(*T*) = CombSet_*τ*_(*T*_all_) (1 ≤ *τ* < *k* − 1). Therefore, for ∀tc ∉ *T*,

(18) MICS(tc,T)=∑τ=1k−1(ωτ×CkτCkτ)+ωk ×|{tc}⋂CombSetk(T)|Ckk=∑τ=1k−1ωτ=1.0−ωk.

In summary, if CombSet_*k*−1_(*T*) = CombSet_*k*−1_(*T*_all_), for ∀tc ∉ *T*, IICS(tc, *T*) = 0 and MICS(tc, *T*) = 1.0 − *ω*_*k*_.

According to Theorem 8, a test case generation method using IICS or MICS as the similarity measure becomes a random generation method, when its generated test suite *T* covers all possible (*k* − 1)-wise value combinations. The main reason is that, for any candidates, no matter whether they are included in *T* or not, the IICS (or MICS) values of all candidates are identical.

**Theorem 9**

If *MICS*(*tc*, *T*) = 0, *IICS*(*tc*, *T*) = 0.

**Proof**

If MICS(tc, *T*) = 0, NCVCS_*τ*_(tc, *T*) = 0 where 1 ≤ *τ* ≤ *k* because of 0 ≤ *ω*_*τ*_ ≤ 1.0 and 0 ≤ NCVCS_*τ*_(tc, *T*) ≤ 1.0; that is, all possible *τ*-wise value combinations covered by tc are not covered by *T*. According to (15), therefore, IICS(tc, *T*) = 0.

As discussed before, both IICS and MICS consider different interaction coverage when evaluating combinatorial test cases. However, they have some differences. Given a combinatorial test case (tc), its IICS measure is actually calculated by the NCVCS_*λ*_ at an appropriate *λ* value, which means that the IICS measure of tc only considers single interaction coverage, while its MICS measure considers different coverage at the same meanwhile. In other words, tc's calculation time of the IICS measure is less than that of the MICS.

In summary, two new similarity measures based on interaction coverage (IICS and MICS) fundamentally differ from NCVCS due to the following reasons: (1) they do not require setting the strength value in advance, and (2) they are more suitable for adaptive strategies than NCVCS.

## 4. Adaptive Random Testing for Combinatorial Test Inputs 

In this section, we propose a new family of methods adopting ART in combinatorial input domain, namely, ART-CID. Similar to previous ART studies, ART-CID can also be implemented according to different notions. In this paper, we present one version of ART-CID by similarity (denoted as FSCS-CID), which uses the strategy of FSCS-ART [15]. Since the similarity measure is used in this paper, the procedure of FSCS-CID may differ from that of FSCS-ART. Detailed information will be given as follows.

### 4.1. Similarity-Based Test Case Selection in FSCS-CID

FSCS-CID uses two test sets, that is, the candidate set *C* of fixed size *l* and the executed set *E*, each of which has the same definition as FSCS-ART. However, test cases in either *C* or *E* are obtained from the combinatorial input domain. For ease of description, let *E* = {*e*_1_, *e*_2_,…, *e*_*m*_} while *C* = {*c*_1_, *c*_2_,…, *c*_*l*_}. In order to select the next test case *c*_*h*_ from *C*, the criterion is described as follows:

(19) (∀ci)(ci∈C) (i=1,2,…,l,i≠h)[max⁡j=1mS(ch,ej)≤ max⁡j=1mS(ci,ej)],

where *S* is the similarity measure between two combinatorial test inputs. The detailed algorithm of implementing (19) is illustrated as follows (see Algorithm 1).

### 4.2. Algorithm of FSCS-CID

As discussed before, Algorithm 1 is used to guide the selection of the best test case. In FSCS-CID, the process of Algorithm 1 runs until the stop condition is satisfied. In this paper, we consider two stop conditions: (1) the first software failure is detected (denoted  StopCon1); and (2) all possible value combinations at strength *τ* are covered (denoted  StopCon2). Detailed algorithm of FSCS-CID is shown in Algorithm 2.

Since the frequencies of parameter values are used in some similarity measures such as* Lin*,* OF*, and* Goodall2*, there requires a fixed-size set of test cases in order to count the frequencies. However, the executed set *E* is incrementally updated with the selected element from the candidate set *C* until the  StopCon1 (or  StopCon2) is satisfied. In this paper, we take the following strategy to construct the fixed-size set of test cases when calculating the similarity between test inputs. During the process of choosing the *i*th (*i* > 1) test input from *C* as the next test case (i.e., |*E* | = *i* − 1), each candidate *c*_*h*_(*c*_*h*_ ∈ *C*) requires to be measured against all elements in *E* according to the similarity measure, and the fixed-size set of test case for *c*_*h*_ is constructed by *E*⋃{*c*_*h*_}.

## 5. Experiment

In this section, some experimental results, including simulations and experiments against real programs, were presented to analyze the effectiveness of FSCS-CID. We mainly compared our method to RT in terms of failure-detection effectiveness (*F*-measure) and the rate of value combinations coverage at a given strength (*N*_*τ*_-measure). For ease of describing our work clearly, we used the terms  Goodall1,  Goodall2,  Goodall3,  Goodall4,  Lin, Lin1,   Overlap,  Eskin,  OF,  IICS,  and  MICS to, respectively, represent the similarity measure* Goodall1*,* Goodall2*,* Goodall3*,* Goodall4*,* Lin*,* Lin1*,* Overlap*,* Eskin*,* OF*, IICS, and MICS adopted in the FSCS-CID. Additionally, we used the term RT to represent RT.

As shown in (16), a weight is required to be assigned for interaction coverage at each strength value. There are many techniques which conduct on assigning weights; however, in this paper we focus on two distribution styles: (1) equal distribution where each interaction coverage has the same weight, that is, *ω*_1_ = *ω*_2_ = ⋯ = *ω*_*k*_ = 1/*k*; and (2) FTFI percentage distribution where according to previous studies [28, 34], for example, in [28], Kuhn et al. investigated several software projects and concluded that the interaction faults are summarized to have 29% to 82% faults as 1-wise faults (i.e., the FTFI number is 1), 6% to 47% of faults as 2-wise faults, 2% to 19% as 3-wise faults, 1% to 7% of faults as 4-wise faults, and even fewer failures beyond 4-wise interactions. As a consequence, we arrange weights as follows: *ω*_1_ = *ω*, *ω*_*i*+1_ = (1/2)*ω*_*i*_, where *i* = 1,2,…, *k* − 1. For example, if *k* = 2, *ω*_1_ = 0.67 and *ω*_2_ = 0.33; if *k* = 3, *ω*_1_ = 0.57, *ω*_2_ = 0.29, and *ω*_3_ = 0.14. In this paper, therefore, we use the terms  MICS1 and  MICS2 to stand for the  MICS techniques with the above two weight distribution styles, respectively.

### 5.1. Simulation

In the following subsection, two simulations were presented to analyze the effectiveness of FSCS-CID according to the rate of covering *τ*-wise value combinations (i.e., *N*_*τ*_-measure). We used two usual test profiles TP(10, 2^10^) and TP(10, 2^5^3^5^) that are commonly used in previous studies [37].

#### 5.1.1. Setup

Since the *τ* was known before testing, in this simulation, we considered the FSCS-CID using NCVCS_*τ*_ [36] as the similarity measure (denoted  NCVCS). Except the  MICS, all other methods do not require to be set. As for the  MICS, different strength values from 1 to *k* are considered to calculate the MICS measure according to (16). However, due to the known *τ*, we mainly focused on the strength values from 1 to *τ* for calculating the MICS measure. As a consequence, (16) becomes as follows:

(20) $$\begin{array}{c} \text{MICS}\left(\text{tc},T\right)=\overset{\tau }{\underset{\lambda =1}{\sum }}\left(\omega_{\lambda }*\text{NCVC}{\text{S}}_{\lambda }\left(\text{tc},T\right)\right), \end{array}$$

where only *ω*_1_, *ω*_2_,…, *ω*_*τ*_ are considered. Each method runs until the  StopCon2 is satisfied. Additionally, we consider *N*_*τ*_ as the metric to evaluate each method in terms of the rate of covering value combinations at strength *τ* for each method, where *τ* = 2,3, 4.

#### 5.1.2. Results

**Figure 3:**
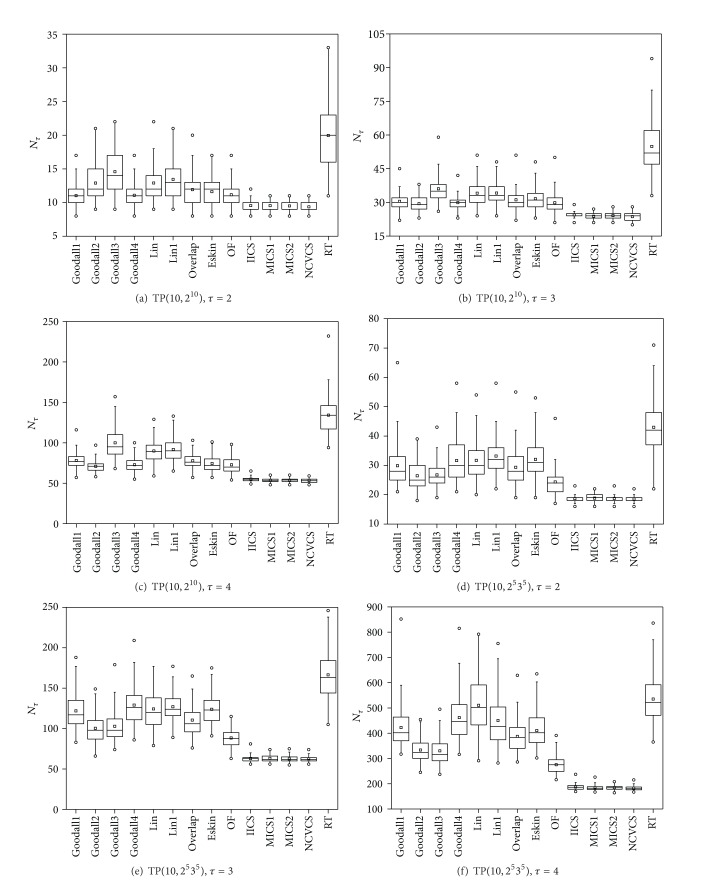
The *N*_*τ*_ metric for different test case generation techniques.

Figure 3 summarizes the number of test cases required to cover all possible *τ*-wise value combinations (i.e., *N*_*τ*_) generated by each method for the above two designed test profiles. Based on the experimental data, we have the following observations.

1. For each test profile, the *N*_*τ*_ (*τ* = 2,3, 4) metric values of all FSCS-CID methods using different similarity measures are smaller than those of RT. In other words, the FSCS-CID methods require the smaller number of test cases for covering all *τ*-wise value combinations than RT, which means that the FSCS-CID methods have the higher rates of covering value combinations than RT.
2. Among all the FSCS-CID methods, the  NCVCS is the most effective technique. The results show that the *N*_*τ*_ values of the  NCVCS are about 30%~50% of those of the  RT. The  IICS has the second best *N*_*τ*_ metric values, followed by the  OF. For TP(10, 2^10^), the  Goodall3 is least effective, while for TP(10, 2^5^3^5^), the Lin performs least.
3. From the perspective of the similarity category, the FSCS-CID methods using the interaction-coverage-based similarity measures (including  IICS,  MICS, and  NCVCS) perform best, while the FSCS-CID methods using the information-theoretic similarity measures (including  Lin and  Lin1) perform worst.

#### 5.1.3. Analysis

Here, we briefly analyze the above observations. The observation (1) is explained as follows. The FSCS-CID methods using different similarity measures select the next test case that has the smallest similarity value against already generated test cases, while RT simply generates teat cases at random from combinatorial input domain. As a consequence, the FSCS-CID methods achieve test cases more diversely than RT over the combinatorial input domain.

As for the observations (1) and (2), they are easy to be explained. On the one hand, since the *N*_*τ*_ metric is related to *τ*-wise value combinations, the  NCVCS performs best because it selects the next test case that covers of uncovered *τ*-wise value combinations as much as possible. In other words, it may have the fastest rate of covering all *τ*-wise value combinations. On the other hand, another two interaction-coverage-based methods, such as IICS and MICS, consider different strength values for generating test cases; however, both of them take the strength *τ* as an indispensable part. In detail, the  IICS calculates the test candidate from the strength 1 to *τ*, while the  MICS considers different strengths from 1 to *τ* at the same time. Hence, it is reasonable that, compared to other categories, the FSCS-CID methods using interaction-coverage-based similarity measures perform best according to the *N*_*τ*_ metric.

### 5.2. An Empirical Study

In this section, an empirical study was conducted to compare the performance between FSCS-CID and RT in practical situations, using the *F*-measure as the effectiveness metric. To describe data clearly, we used ART *F*-ratio, which is defined as the *F*-measure ratio between FSCS-CID and RT, that is, *F*_FSCS-CID_/*F*_RT_. Intuitively speaking, the smaller ART *F*-ratio value implies the higher improvement of FSCS-CID over RT, and (1 − *F*_FSCS-CID_/*F*_RT_) is the *F*-measure improvement of FSCS-CID over RT.

In this empirical study, we use a set of six fault-seeded C programs with 9 versions. The five subject programs, including  count,  series,  tokens,  ntree, and  nametbl, are downloaded from Chris Lott's website (http://www.maultech.com/chrislott/work/exp/), which have been widely used in the research of combinatorial space such as comparison of defect revealing mechanisms [38], evaluation of different combination strategies for test case selection [39], and fault diagnosis [40, 41]. The remainder subject programs are a series of   flex programs (the model used in this paper is unconstrained, which has some limitations: “We note that in a real test environment an unconstrained TSL would most likely be prohibitive in size and would not be used” [42].), downloaded from Software Infrastructure Repository (SIR) [43], which are popularly used in combinatorial test suite construction [44] and combinatorial interaction regression testing [42].

Table 3 presents detailed information about these subject programs, from which the third column “LOC” represents the number of lines of executable code in these programs, and “#S.” is the number of seeded faults in each subject program, while “#D.” is the number of faults that can be detected by some test cases derived from the accompanying test profiles, which are not guaranteed to be able to detect all faults. However, in our study, we only use a portion of detectable faults, of which the size is shown as “#U.”. The main reason is due to the fact that faults in the set of detectable faults but not in the set of used faults have high failure rates that exceed 0.5. As we know, if the failure rate *θ* of a fault is larger than 0.5, the *F*-measure of random testing is theoretically less than 1/*θ* = 2. As a consequence, the *F*-measure of FSCS-CID depends on the first randomly selected test case. In other words, if the first test case cannot detect a failure, the *F*_FSCS-CID_ is larger than or equal to 2. Therefore, the *F*-measure of FSCS-CID is dependent on random testing.

For the purpose of clear description, we order *i* used faults in each subject program in a descend order according to failure rate and abbreviate them as *m*_1_, *m*_2_,…, *m*_*i*_. The range of failure rates in each program, as shown in Table 3, is from *θ*_*S*_ to *θ*_*L*_.

**Figure 4:**
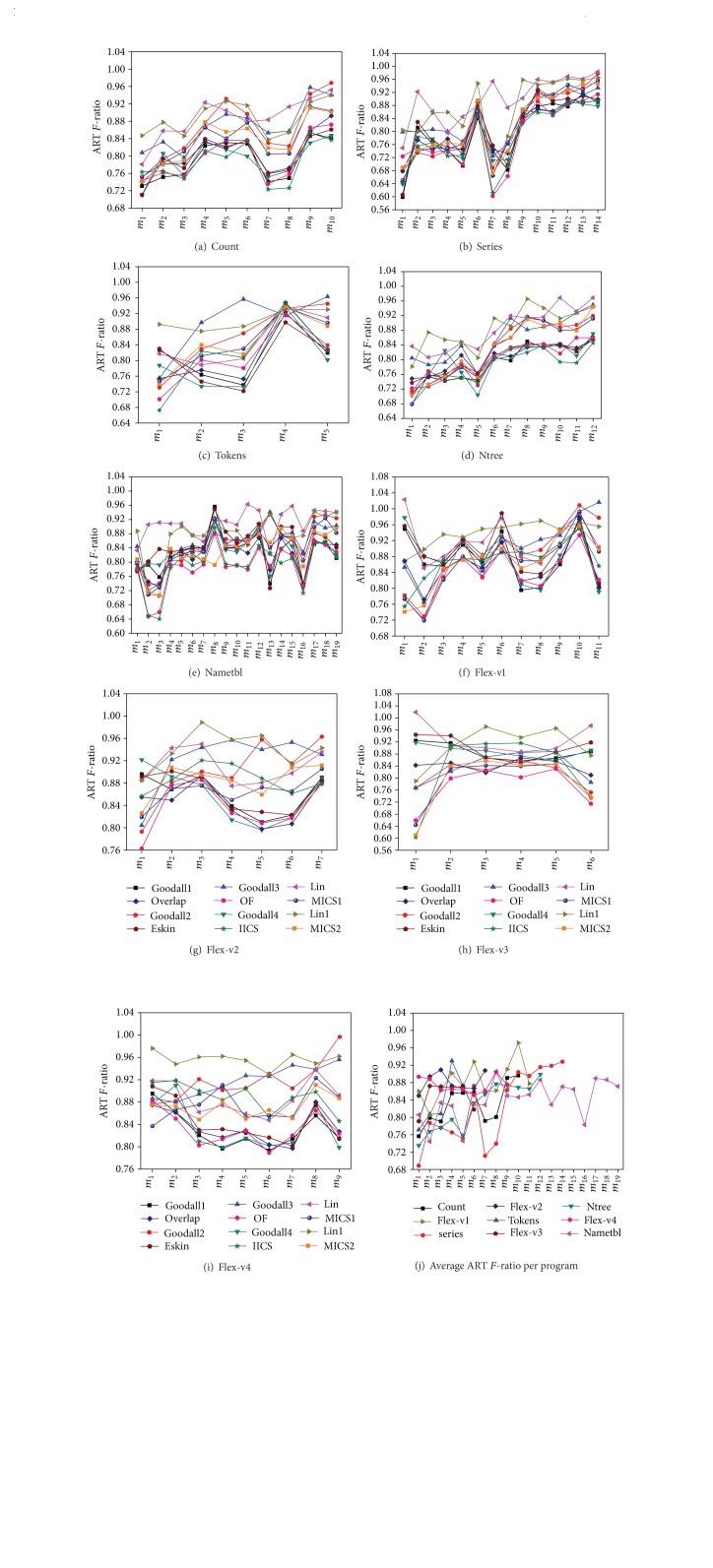
ART *F*-ratio of FSCS-CID methods using different similarity measures.

We used all twelve FSCS-CID versions using different similarity measures to test these fault-seeded programs. The results of the empirical study are given in Figure 4, where *x*-axis represents each seeded fault in the subject program, while *y*-axis represents the ART *F*-ratio. As shown in Figures 4(a)–4(i), each figure corresponds to a particular subject program, while Figure 4(j) represents the average ART *F*-ratio of all FSCS-CID versions for each subject program.

From Figures 4(a)–4(j), we can observe the following conclusions.

1. According to ART *F*-ratio, all twelve FSCS-CID versions, including  Goodall1,  Goodall2,  Goodall3,  Goodall4,  Lin,  Lin1,  Overlap,  Eskin,  OF,  IICS,  MICS1, and  MICS2, perform better than  RT. In the best case, the improvement of FSCS-CID over RT is about 40% (i.e., ART *F*-ratio is 60%).
2. With the increase of failure rate *θ*, the ART *F*-ratio of each FSCS-CID version increases as well in most programs. In other words, when *θ* is larger, the improvement of each FSCS-CID version over RT is smaller.
3. The failure-detection capability of FSCS-CID depends on some factors, such as the program (or test profile) and failure type (including failure rate *θ* and failure pattern). For example, in program count faults *m*_3_ and *m*_4_ have the same failure rate; however, the ART *F*-ratio of each FSCS-CID version when detecting *m*_3_ is very different from that when detecting *m*_4_.
4. Figure 4(j) describes the average ART *F*-ratio of all FSCS-CID versions when detecting each fault for each subject program. It can be clearly seen that the ART *F*-ratio of the FSCS-CID algorithm generally fluctuates from 0.75 to 0.90 among all faults for each program, which means FSCS-CID can improve about 10%~25% of *F*-measure over RT in the average.
5. Among all FSCS-CID versions, no method performs best for all programs, and no method performs worst. In order to compare the failure-detection capabilities of different FSCS-CID versions, Table 4 shows the average ART *F*-ratio of each FSCS-CID version for each subject program. According to data shown in Table 4, it is obvious that in general one of FSCS-CID version  OF performs best, followed by  IICS, while  Lin and  Lin1 generally perform worst. In addition,   Eskin performs best for the program  tokens and  Goodall1 has the best performance for the program  flex-v4.

In summary, our simulation results (Section 5.1) have shown that our FSCS-CID algorithm (irrespective of used similarity measure) has higher rates of covering value combinations at different strength values than those of random testing. Besides, the empirical study has shown that the FSCS-CID algorithm performs better than RT in terms of the number of test cases required to detect the first failure (i.e., *F*-measure).

### 5.3. Threats to Validity

The experimental results suffer from some threats to validity; in this section, we outline the major threats. In the simulation study, two widely used, but limited, test profiles were employed. In the empirical study, many real-life programs were used, which have been popularly investigated by different researches. However, the faults seeded in each subject program have high failure rates. To address these potential threats, additional studies using a great number of test profiles and a great number of subject programs with low failure rates will be investigated in the future.

In addition, although two metrics (*N*_*τ*_-measure and *F*-measure) were employed in our experiment, we recognize that there may be other metrics which are more pertinent to the study.

## 6. Discussion and Conclusion 

Adaptive random testing (ART) [15] has been proposed to enhance the failure-detection capability of random testing (RT) by evenly spreading test cases all over the input domain and has been widely applied in various applications such as numerical programs, Java programs, and object-oriented programs. In this paper, we broaden the principle of ART in a new type of input domain that has not yet been investigated, that is, combinatorial input domain. Due to special characteristics of combinatorial input domain, the test case similarity (or dissimilarity) measures previously used in ART may not be suitable for combinatorial input domain. By adopting some well-known similarity measures used in data mining and proposing two new similarity measures based on interaction coverage, we proposed a new approach to apply original ART into combinatorial input domain, named ART-CID. We conducted some experiments including simulations and the empirical study to analyze the effectiveness of one version of ART-CID (FSCS-CID, which is based on fixed-size-candidate-set ART). Compared with RT, FSCS-CID not only brings higher rates in covering all possible combinations at any given strengths, but also requires fewer combinatorial test cases to detect the first failure in the seeded program.

Combinatorial interaction testing (CIT) [33] is a black-box testing method and has been widely used in combinatorial input domain. It aims at constructing an effective test suite to identify interaction faults caused by parameter interactions. Some greedy CIT algorithms, such as AETG [45], TCG [46], and DDA [47], may have similar mechanism as FSCS-CID. Taking AETG for example, similar to AETG, FSCS-CID also first constructs some candidates, and then from which the “*best*” element would be chosen as the next test case according to some criteria. However, there are some fundamental differences between AETG and FSCS-CID, which are mainly summarized as follows.

1. Different construction strategies of candidates: FSCS-CID constructs candidates in a random manner, while AETG first orders all parameters and then assigns a value to each parameter, such that all assigned parameter values can cover the largest number of value combinations at a given strength.
2. Different test case selection criteria: AETG selects an element from candidates as the next test case such that it covers the largest number of value combinations at a given strength, while FSCS-CID chooses the next test case according to its used similarity measure.
3. Different goals achieved: AETG aims at covering all possible value combinations of a given strength with fewer test cases, which means that the unique stopping condition of AETG is that all value combinations of a given strength are covered by generated test cases, while FSCS-CID is an adaptive strategy, which means that the stopping condition of FSCS-CID is not limited to covering all value combinations of a give strength, for example, detecting a first failure in the SUT.

In this paper, constraints among value combinations have not been considered; however, they often exist in real-life programs. For example, as shown in Table 1, there may exist a constraint among “*Full Size*” of *p*_1_ and “*Single*” of *p*_2_, that is, when *p*_1_ = *Full* *Size*, *p*_2_ ≠ *Single* (i.e., “*Full Size*” and “*Single*” cannot occur in a combinatorial test cases). In this case, the method FSCS-CID proposed in this paper can still be successfully executed only by judging that each selected test case violates constraints among value combinations or not. Generally speaking, this judgment process can be implemented in the following phases: (1) when constructing the candidate set and (2) when adding the latest test case into the executed set. However, how to deal with constraints among value combinations should be further studied.

In the future, we plan to further investigate how to improve the effectiveness of the approach by adopting other similarity measures that may be available in combinatorial input domain or by considering additional factors to guide test case generation. In addition, how to extend other original ART algorithms into combinatorial input domain is also expected.

## Figures and Tables

**Algorithm 1:**
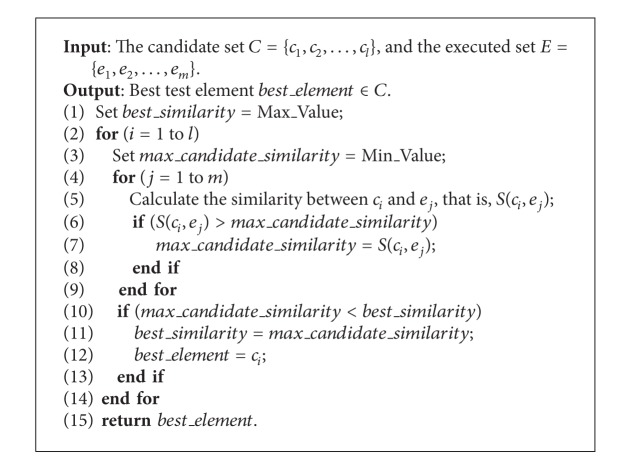
Select the best test element based on similarity (BTES).

**Algorithm 2:**
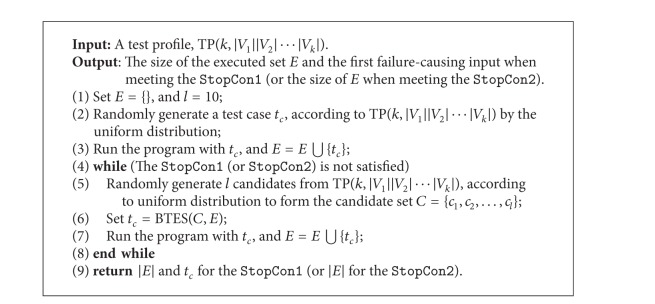
The algorithm of FSCS-CID.

**Table 1:** Optional parameters and values of an option “View" in the tool PDF.

Parameters	*p*_1_: Page size	*p*_2_: Page layout	*p*_3_: Rotating view	*p*_4_: Reading
Values	*Full size *	*Single *	*Clockwise *	*Current Page *
	*Right width *	*Bisected *	*Anticlockwise *	*All pages *
	*Right page *	*Continuous *	*None *	*None *

**Table 2:** Similarity measures for categorical parameter values.

Measure	*S*_*i*_(*X*_*i*_, *Y*_*i*_)	*ω*_*i*_, *i* = 1,2,…, *k*	Range
*Goodall1 *	$=\{\begin{array}{cc} 1-\underset{q\in Q}{\sum }‍p_{i}^{2}(q) & \text{if}\;\;X_{i}=Y_{i}\\ 0 & \text{otherwise} \end{array}$	$\frac{1}{k}$	$\left[0,1-\frac{2}{N^{2}}\right]$ [0,0]

*Goodall2 *	$=\{\begin{array}{cc} 1-\underset{q\in Q}{\sum }‍p_{i}^{2}(q) & \text{if}\;{\;X}_{i}=Y_{i}\\ 0 & \text{otherwise} \end{array}$	$\frac{1}{k}$	$\left[1-\frac{2}{N^{2}},1\right]$ [0,0]

*Goodall3 *	$=\{\begin{array}{cc} 1-\sum ‍p_{i}^{2}(X_{i}) & \text{if}{\;\;X}_{i}=Y_{i}\\ 0 & \text{otherwise} \end{array}$	$\frac{1}{k}$	$\left[0,1-\frac{1}{N^{2}}\right]$ [0,0]

*Goodall4 *	$=\{\begin{array}{cc} \sum ‍p_{i}^{2}(X_{i}) & \text{if }\;X_{i}=Y_{i}\\ 0 & \text{otherwise} \end{array}$	$\frac{1}{k}$	$\left[\frac{1}{N^{2}},1\right]$ [0,0]

*Lin *	$=\{\begin{array}{cc} 2\log{\hat{p}}_{i}(X_{i}) & \text{if}{\;\;X}_{i}=Y_{i}\\ 2\log\;({\hat{p}}_{i}(X_{i})+{\hat{p}}_{i}(Y_{i})) & \text{otherwise} \end{array}$	$\frac{1}{\sum_{i=1}^{k}‍\log{\hat{p}}_{i}(X_{i})+\log{\hat{p}}_{i}(Y_{i})}$	[−2log⁡⁡N, 0] $\left[-2\log\frac{N}{2},0\right]$

*Lin1 *	$=\{\begin{array}{cc} \underset{q\in Q}{\sum }‍\log{\hat{p}}_{k}(q) & \text{if}\;\;X_{i}=Y_{i}\\ 2\log\underset{q\in Q}{\sum }‍{\hat{p}}_{k}(q) & \text{otherwise} \end{array}$	$\frac{1}{\sum_{i=1}^{k}‍\sum_{q\in Q}‍\log{\hat{p}}_{i}(X_{i})}$	[−*N*log⁡⁡*N*, 0] $\left[-2\log\frac{N}{2},0\right]$

*Overlap *	$=\{\begin{array}{cc} 1 & \text{if}{\;\;X}_{i}=Y_{i}\\ 0 & \text{otherwise} \end{array}$	$\frac{1}{k}$	[1,1] [0,0]

*Eskin *	$=\{\begin{array}{cc} 1 & \text{if}{\;\;X}_{i}=Y_{i}\\ \frac{{\left|V_{i}\right|}^{2}}{{\left|V_{i}\right|}^{2}+2} & \text{otherwise} \end{array}$	$\frac{1}{k}$	[1,1] $\left[\frac{2}{3},\frac{N^{2}}{N^{2}+2}\right]$

*OF *	$=\{\begin{array}{cc} 1 & \text{if}{\;\;X}_{i}=Y_{i}\\ \frac{1}{1+\log\left(N/f_{i}(X_{i})\right)\times \log\left(N/f_{i}(Y_{i})\right)} & \text{otherwise} \end{array}$	$\frac{1}{k}$	[1,1] $\left[\frac{1}{1+{\left(\log N\right)}^{2}},\frac{1}{1+{\left(\log2\right)}^{2}}\right]$

Note. For measure *Goodall1*, *Q* = {*q* | *q* ∈ *V*_*i*_, *p*_*i*_(*q*) ≥ *p*_*i*_(*X*_*i*_)}.

For measure *Goodall2*, *Q* = {*q* | *q* ∈ *V*_*i*_, *p*_*i*_(*q*) ≤ *p*_*i*_(*X*_*i*_)}.

For measure *Lin1*, $Q=\{q|q\in V_{i},\min({\hat{p}}_{i}(X_{i}),{\hat{p}}_{i}(Y_{i}))\leq {\hat{p}}_{i}(q)\leq \max\left({\hat{p}}_{i}(X_{i}),{\hat{p}}_{i}(Y_{i}))\right\}$.

**Table 3:** The detailed information about 6 programs with 9 versions.

Program	Test profile	LOC	#S.	#D.	#U.	*θ*_*S*_	*θ*_*L*_
Count	TP(6, 2^1^3^5^)	42	15	12	10	0.111111	0.444444
Series	TP(3, 5^2^7^1^)	288	23	22	14	0.034286	0.462857
Tokens	TP(8, 2^4^3^4^)	192	21	15	5	0.111111	0.388889
Ntree	TP(4, 4^4^)	307	32	24	12	0.023438	0.492188
Nametbl	TP(5, 2^1^3^2^5^2^)	329	51	44	19	0.133333	0.444444

Flex-v1	TP(7, 2^4^3^1^6^1^16^1^)	8426	19	16	11	0.031250	0.427083
Flex-v2		9932	20	11	7	0.019097	0.375000
Flex-v3		9965	17	6	6	0.020833	0.118056
Flex-v4		10055	16	11	9	0.135417	0.427083

**Table 4:** The average ART F*-*ratio of each FSCS-CID version for each subject program (%).

Method	Count	Series	Tokens	Ntree	Nametbl	Flex-v1	Flex-v2	Flex-v3	Flex-v4
G oodall1	79.07^a^	79.75	81.38	79.24	82.76	87.49	85.99	88.65	**82.97**
Goodall2	86.31	81.84	86.16	83.59	85.17	88.47	89.99	81.38	91.80
Goodall3	86.95	84.17	89.74	85.72	85.54	90.86	92.16	83.66	91.80
Goodall4	79.15	79.58	79.86	79.60	82.66	87.84	85.81	88.86	83.28
Lin	*88.84* ^ b^	*90.07 *	85.18	88.57	*90.97 *	92.21	90.94	*93.10 *	88.79
Lin1	88.79	87.65	*90.31 *	*88.81 *	87.57	*93.95 *	*94.06 *	90.63	*95.64 *
Overlap	80.76	81.01	81.11	80.56	82.36	85.64	84.53	84.06	83.53
Eskin	80.07	81.32	**80.47**	80.39	84.65	89.35	86.42	90.09	84.35
OF	79.70	78.84	80.80	79.75	**79.09**	**83.57**	**83.64**	**77.20**	83.07
IICS	78.59	**78.72**	81.59	**78.27**	79.29	87.06	88.67	82.69	88.97
MICS1	83.58	81.77	84.43	82.63	84.21	86.02	86.53	79.00	87.37
MICS2	83.55	82.30	84.42	83.05	83.12	86.56	88.47	78.89	87.11

^a^The bold datum is minimum in each column.

^b^The italic datum is maximum in each column.
